# Arbuscular Mycorrhizal Fungus Species Dependency Governs Better Plant Physiological Characteristics and Leaf Quality of Mulberry (*Morus alba* L.) Seedlings

**DOI:** 10.3389/fmicb.2016.01030

**Published:** 2016-06-28

**Authors:** Song-Mei Shi, Ke Chen, Yuan Gao, Bei Liu, Xiao-Hong Yang, Xian-Zhi Huang, Gui-Xi Liu, Li-Quan Zhu, Xin-Hua He

**Affiliations:** ^1^Key Laboratory of Horticulture Science for Southern Mountainous Regions, Ministry of Education/College of Horticulture and Landscape Architecture, Southwest UniversityChongqing, China; ^2^Centre of Excellence for Soil Biology, College of Resources and Environment, Southwest UniversityChongqing, China; ^3^State Key Laboratory of Silkworm Genome Biology, Southwest UniversityChongqing, China; ^4^School of Plant Biology, University of Western Australia, CrawleyWA, Australia

**Keywords:** *Acaulospra scrobiculata*, amino acids, *Funneliformis mosseae*, *Rhizophagus intraradices*, net leaf photosynthesis rate, transpiration

## Abstract

Understanding the synergic interactions between arbuscular mycorrhizal fungi (AMF) and its host mulberry (*Morus alba* L.), an important perennial multipurpose plant, has theoretical and practical significance in mulberry plantation, silkworm cultivation, and relevant textile industry. In a greenhouse study, we compared functional distinctions of three genetically different AMF species (*Acaulospora scrobiculata*, *Funneliformis mosseae*, and *Rhizophagus intraradices*) on physiological and growth characteristics as well as leaf quality of 6-month-old mulberry seedlings. Results showed that mulberry was AMF-species dependent, and AMF colonization significantly increased shoot height and taproot length, stem base and taproot diameter, leaf and fibrous root numbers, and shoot and root biomass production. Meanwhile, leaf chlorophyll a or b and carotenoid concentrations, net photosynthetic rate, transpiration rate and stomatal conductance were generally significantly greater, while intercellular CO_2_ concentration was significantly lower in AMF-inoculated seedlings than in non-AMF-inoculated counterparts. These trends were also generally true for leaf moisture, total nitrogen, all essential amino acids, histidine, proline, soluble protein, sugar, and fatty acid as they were significantly increased under mycorrhization. Among these three tested AMFs, significantly greater effects of AMF on above-mentioned mulberry physiological and growth characteristics ranked as *F. mosseae* > *A. scrobiculata* > *R. intraradices*, whilst on mulberry leaf quality (e.g., nutraceutical values) for better silkworm growth as *F. mosseae* ≈*A. scrobiculata* > *R. intraradices*. In conclusion, our results showed that greater mulberry biomass production, and nutritional quality varied with AMF species or was AMF-species dependent. Such improvements were mainly attributed to AMF-induced positive alterations of mulberry leaf photosynthetic pigments, net photosynthetic rate, transpiration rate, and N-containing compounds (methionine, threonine, histidine, and proline). As a result, application of *Funneliformis mosseae* or *A. scrobiculata* in mulberry plantation could be a promising management strategy to promote silkworm cultivation and relevant textile industry.

## Introduction

As a multipurpose plant, mulberry (*Morus alba* L.) has become an increasingly attractive plant for its economic value and restoration function. It has been extensively cultivated in China for the main purpose of rearing silkworm (*Bombyx mori* L.), an economically important insect as the primary producer of silk, and thus contributing to local farming and textile industries. The quality of mulberry leaves strongly associates with the quality and quantity of cocoon, especially their high contents of carbon, nitrogen (N), amino acids and proteins that contribute greatly to the silkworm development (Machii and Katagiri, 1991; Sujathamma and Dandin, 2000; Rao et al., 2007). Although mulberry can survive in poor soil (Kumar et al., 2003; Huang et al., 2013), but its growth is retarded under infertile soil. A number of biotic or abiotic factors, such as soil beneficial organisms and fertilization, etc., do enhance the growth and leaf quality of mulberry (Jeffries et al., 2003; Setua et al., 2007; Chowdhury et al., 2013; Fernandez et al., 2014).

The symbiotic associations formed by arbuscular mycorrhizal fungi (AMF) are widely associated with >80% vascular plants including mulberry in terrestrial ecosystems (Smith and Read, 2008). Rajagopal et al. (1989) was probably the first to report that mulberry roots were highly colonized by AMF in natural conditions, and phosphorus (P) uptake was hence increased to lead a better leaf growth and biomass production of mulberry (Katiyar et al., 1995; Setua et al., 1999a,b). Both the growth and yield of 10-year-old mulberry plants were similar between fertilizations under 100% P and 50% P + *Glomus* (now *Funneliformis*) *mosseae* inoculation (Mamatha et al., 2002). Recently, for 90-day-old AMF inoculated mulberry seedlings, either *Funneliformis mosseae* or *Glomus* (now *Rhizophagus*) *intraradices* had enhanced leaf numbers, plant height, chlorophyll, N and P, both aboveground and belowground biomass production, root length and root TTC (2,3,5-triphenyl tetrazolium chloride) deoxidizing ability, although there was no such growth benefits by their dual fungal inoculation (Lu et al., 2015). Meanwhile, the combined application of mycorrhizal fungi and other beneficial microorganisms could also promote the growth of mulberry. For instance, the co-inoculation of two AMF species (*Glomus fasciculatum* and *F. mosseae*) with other three beneficial microorganisms (*N*_2_-fixing *Azotobacter* sp., phosphate solubilizing bacterium *Bacillus megaterium* and fungus *Aspergillus awamori*) did increase the uptake of N, P, K (potassium) and leaf chlorophyll in 1-year-old mulberry plants in India (Baqual and Das, 2006). In addition, a combined positive effect of *F. mosseae* and bacterial biofertilizer (*Azotobacter chrococcum*) on mulberry leaf quality and cocoon characters was also found under semiarid fields in India (Rao et al., 2007). However, limited information is available about the influence of different AMF species either within the same fungal genus or different genera on the quantity and quality of mulberry leaves, which in turn will directly affect the growth and development of silkworm, the production of cocoon and the quality of silk products. Therefore, further studies will determine if different AMF species in the same genus or different genera have distinctive roles in the growth and leaf quality of mulberry.

We have found that a high diversity of AMF, including *Acaulospra scrobiculata, Funneliformis mosseae*, and *Rhizophagus intraradices*, associates with field-growing mulberry plants, and that such an individual AM fungus promotes the growth of mulberry (Shu et al., 2011; Shi et al., 2013) and soil fertility (Chen et al., 2014) in southwest China. In this study, we hypothesized that the growth of mulberry and quality of mulberry leaves could have varied responses to different AMF species or their physiological characteristics of mulberry might be AMF-species dependent. Our objectives were to address the following questions: (1) If AM fungus could successfully colonize the host mulberry, and what physiological alterations could the host have after colonization? (2) Whether the growth and performance of mulberry depend on its symbiont fungal species characteristics or not? In doing so, 3-month-old mulberry seedlings grown in a greenhouse were inoculated with or without an individual AMF species (*A. scrobiculata, F. mosseae*, and *R. intraradices*) from three different AM genera, and some of their basic physiological characteristics were then compared after a further 3-month-old growth. Answers to these questions could improve our understanding of the physiological responses of mulberry to different AM fungi in order to employ better functionally effective AM species for promoting sustainable mulberry plantation and silkworm industry in China.

## Materials and Methods

### Experimental Design

The greenhouse experiment set-up was a randomized complete block design that had three AMF inoculation treatments (*Acaulospra scrobiculata, Funneliformis mosseae*, or *Rhizophagus intraradices*) and a non-AMF control with four replicates for each treatment.

### Plants, Mycorrhizal Inoculums, and Plant Growth Media

Seeds of mulberry (*Morus alba* var. Gui-you-sang 12, supplied by the Institute of Sericulture and Systems Biology, Southwest University, Chongqing, China) were disinfected with 0.1% HgCl_2_ for 5 min, rinsed thoroughly with sterilized water and then germinated on sterilized moistened filter paper. The germinated seeds were cultivated for 1 month in plastic trays, which contained autoclaved (121°C, 0.1 MPa, 120 min) sand growth media (peat: decomposed rice chaff: sand = 2:2:1, v/v/v.

Three AMF inoculums [*A. scrobiculata* (BGC HK02A), *Funneliformis mosseae* (BGC NM04A) and *R. intraradices* (BGC AH01)] were purchased from the Bank of Glomales in China (BGC), locating in the Institute of Plant Nutrition and Resources, Beijing Academy of Agriculture and Forestry. Inoculums consisted of spores (40–50 spores per 10 g dry soil), mycorrhizal mycelia, root segments, and sand. The growth medium was a mixture of soil (Eutric Regosol, FAO Soil Taxonomic Classification), peat and rice chaff (2:1:1, v/v/v). With a pH 7.28, this soil growth medium had 71.17 g organic matter/kg, 373.12 mg available nitrogen/kg, 14.72 mg available phosphorus/kg, 132.57 mg available potassium/kg.

### Plant Growth and Harvest

Four seedbeds containing the above-mentioned soil growth medium were used to grow mycorrhizal mulberry seedlings. Each seedbed (28 m × 1.2 m) was fumigated with 0.5% formaldehyde and divided into four blocks or replicates (6 m × 1.2 m with 1 m interblock space) for each treatment. A total of 600 1-month-old non-mycorrhizal mulberry seedlings from the sand growth media were transplanted and grown in seedbeds with the autoclaved soil growth media. After 2 months, about 400 uniform seedlings (100 for each block or replicate) were chosen for mycorrhizal inoculation with 3 g AMF inoculum (autoclaved for the control) close to each root rhizosphere. Plants were then grown in a greenhouse under 26/15°C (day/night), 70–80% relative humidity and natural light intensity. No-fertilization was employed for a further 3-month period till harvest (i.e., the total growth period were 6 months).

After 3 months of mycorrhizal inoculation, 20 uniform seedlings out of the 100 seedlings from each replicate were randomly harvested and a total of eighty seedlings were thus harvested from each treatment to determine growth and physiological parameters. At harvest, plant height, stem diameter, leaf numbers, taproot length and diameter, and fibrous root numbers were recorded. Leaves, shoots and roots were then oven-dried at 70°C and weighed. The fresh fifth fully expanded leaves from the top to the bottom of each seedling were harvested, and a total of 80 leaves from each treatment were divided into two sample groups. One sample group of leaves was cut into pieces and mixed for the determination of leaf chlorophyll and carotenoid, soluble protein and sugar (see below). Another sample group of leaves was oven-dried at 70°C till constant weight, pulverized to fine powder for the determination of amino acid, total nitrogen, and fatty acid (see below). The leaf moisture was recorded according to the method described by Gao (2000).

### Determination of Mycorrhizal Colonization and Dependency

Root mycorrhizal colonization was measured according to Phillips and Hayman (1970). The fresh roots were cut into 2 cm segments and cleared with 10% (w/v) KOH at 98°C for 30 min, rinsed in water three times, and bleached with 10% H_2_O_2_ until root clear. The clear root segments were then soaked into 0.2 M HCl for 3 min and stained with 0.05% trypan blue. Five stained segments were mounted on one slide and a total of 50 root segments from each treatment were examined under microscope. The root mycorrhizal colonization was determined as the percent (%) of infected root segments out of the total observed root segments. Mycorrhizal dependency (MD) was calculated as the dry weight of mycorrhizal seedling out of the dry weight of non-mycorrhizal seedling (Menge and Johnson, 1978).

### Photosynthesis Measurements

The fifth leaf from the top to the bottom of each plant growing in the greenhouse was selected for the measurements of photosynthesis variables. Gas exchange parameters (including net photosynthetic rate, stomatal conductance, intercellular CO_2_ concentration, and transpiration rate) were determined with 20 seedlings from each replicate using a portable infrared gas analyzer LI-COR 6400 (LI-COR, Inc., Lincoln, NE, USA) from 09:00 to 11:00 am on a sunny day after 3 months of mycorrhizal inoculation. The photosynthetically active radiation was 1000 ± 12 μmol m^–2^ s^–1^, CO_2_ concentration 350 ± 2 cm^3^ m^–3^, leaf temperature 28.0 ± 0.8°C, and flow rate of atmosphere 0.5 dm^3^ min^–1^. Meanwhile, the chlorophyll and carotenoid in the fresh leaves were extracted with 80% acetone and the absorbance was read spectrophotometrically at 663 and 645 nm. Chlorophyll and carotenoid concentration was estimated by the method of Hiscox and Israelstam (1979).

### Determination of Leaf Soluble Sugar, Soluble Protein, Amino Acid, Fatty Acid, and Total Nitrogen

Determination of soluble sugar (mg g^–1^ FW) in fresh leaves was followed by the anthrone method (Yemm and Willis, 1954). Briefly, 0.5 g fresh leaves were grinded with 80% ethanol and then centrifuged at 3,500 × *g* for 10 min. The supernatant was collected. The total soluble sugar was determined by reacting 0.1 ml supernatant with 5 ml freshly prepared anthrone sulphuric acid solution (75%, v/v), and incubated in boiling water for 10 min. After cooled, the absorbance of the incubated supernatant was spectrophotometrically read at 620 nm.

Soluble proteins (mg g^–1^ FW) of fresh leaves were extracted with 50 mM potassium phosphate buffer (pH 7.5) on ice. The extracts were centrifuged at 12,000 × *g* for 10 min at 4°C. The supernatant was then collected for soluble protein determination according to the protein dye-binding method of Bradford (1976). For each extract, the absorbance was spectrophotometrically read at 595 nm and the bovine serum albumin (BSA) protein was used as the standard.

For the measurement of leaf total amino acids (mg g^–1^ DW), 230 mg leaf powder was put into a beaker containing 8 ml 6 M HCl, and hydrolyzed at 110°C for 22 h in thermostat drier. After cooled, the solution was filtered and the filtrate was transferred into a volumetric flask and dried at 60°C. The dried hydrolysate was dissolved in 0.02 M HCl and then analyzed for total amino acids with the ninhydrin method (Lee and Takahashi, 1966) by an automatic amino acid analyzer (L-8900; Hitachi, Tokyo, Japan).

Leaf fatty acid (mg g^–1^ DW) was based on the Chinese Standard Method (GB5009.6-85). Briefly, 3 g dried leaf powder was placed in a filter cartridge that was dried at 105°C for 2 h. The cartridge was placed in a 50 mL anhydrous ether and refluxed 12 h using a Soxhlet apparatus. After the ether was fully evaporated from the extract, the cartridge containing the extract was oven-dried at 105°C, cooled in a desiccator and then weighed.

The leaf nitrogen was determined with the Kjeldahl procedure. Briefly, 0.5 g leaf powder was placed in a digestion tube containing 10 ml digestion reagent and then dissolved in concentrated H_2_SO_4_. After gently mixed, the digestion tube was placed in the heating block preheated at 300°C. A small glass funnel was inserted in the mouth of the digestion tube for ensuring efficient refluxing of the digestion mixture and preventing loss of H_2_SO_4_. The sample was digested at the boiling point of the mixture for 2.5 h, removed from the heating block, and cooled to room temperature. The digest was diluted with distilled water to 100 ml and performed a steam distillation analysis as described by Nelson and Sommers (1973).

All relevant variables were analyzed with a UV1000 spectrophotometer and all analyses were performed three times.

### Statistical Analyses

Data were subjected to one-way ANOVA and significant differences among treatments were tested by the Duncan’s multiple range test at *P* < 0.05 using the SPSS 19.0 (SPSS Inc., Chicago, IL, USA). To test correlations, a polynomial regression analysis was performed using the OriginPro 8.0 (OriginLab Corp., Northampton, MA, USA).

## Results

### Effects of AMF Colonization on Plant Growth

**FIGURE 1:**
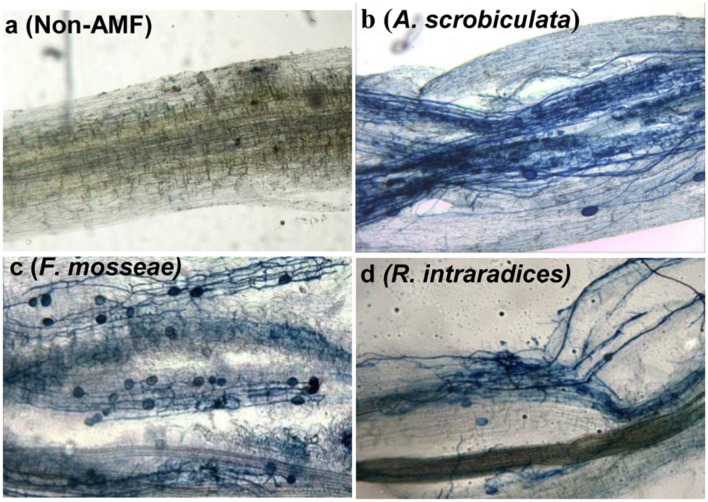
**Root arbuscular mycorrhizal fungal colonization in 6-month-old mulberry seedlings.** Fifty mycorrhizal root segments from each treatment were examined under a microscope: **(a)** non-AMF treatment; **(b)** *A. scrobiculata, Acaulospora scrobiculata*; **(c)** *F. mosseae, Funneliformis mosseae*; **(d)** *R. intraradices, Rhizophagus intraradices*.

**FIGURE 2:**
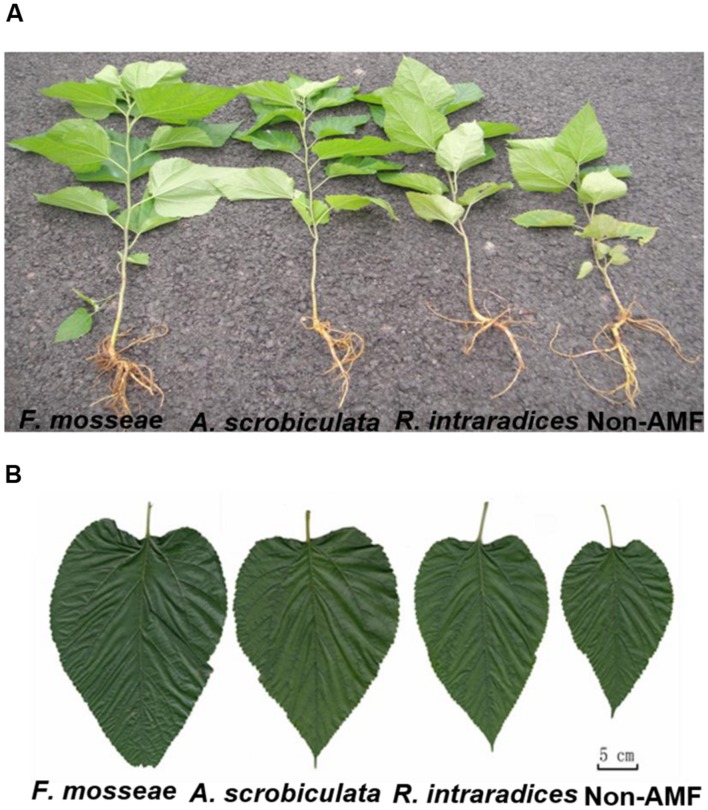
**Plant growth status of 6-month-old mulberry seedlings as influenced by mycorrhizal fungal species. (A)** Whole plant growth, and **(B)** the growth of the fifth fully expanded leaf.

**FIGURE 3:**
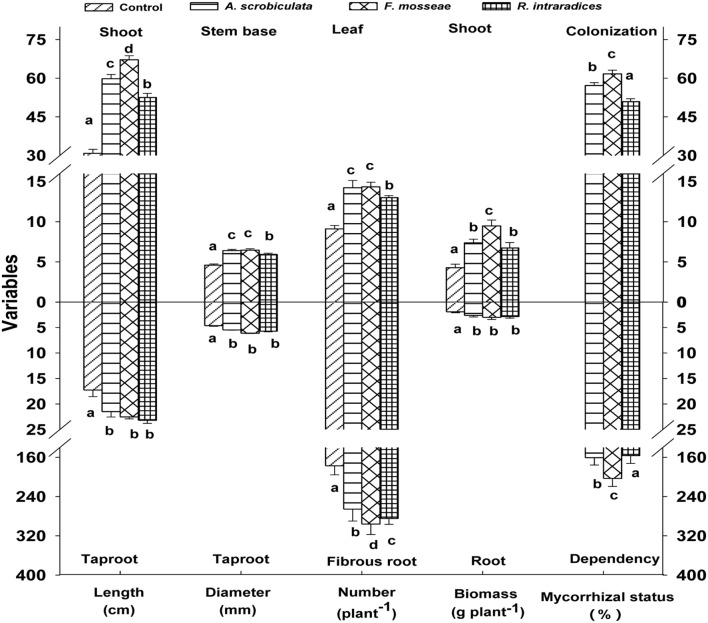
**Effects of mycorrhizal fungal species on growth performance of mulberry plants.** Data (means ± SE, *n* = 4) followed by different letters indicate significant differences among AMF treatments at *P* < 0.05. Eighty plants from each treatment were randomly selected to detect growth performance. Abbreviations: Control, non-AMF treatment; *A. scrobiculata*, *Acaulospora scrobiculata*; *F. mosseae*, *Funneliformis mosseae*; *R. intraradices*, *Rhizophagus intraradices*.

Root AMF colonization existed only in AMF inoculated mulberry plants, and neither AMF colonization nor AMF structures were microscopically observed in the control seedlings (**Figure 1**). Percentages of root colonization in mulberry seedlings were over 50% and significantly highest with *F. mosseae*, greater with *A. scrobiculata* and least with *R. intraradices* (**Figure 1**). The mycelial numbers were similar among the three mycorrhizal inoculations, while the vesicle numbers were lower under the *R. intraradices* treatment (**Figure 1**). Correspondingly, the growth performance of mulberry seedlings was affected by mycorrhizal fungal colonization (**Figure 2**). Compared with non-mycorrhizal seedlings, all tested plant growth variables, including aboveground height and taproot length, stem base and taproot diameter, shoot and root biomass, leaf and fibrous root numbers, were significantly increased by a range of 31 to 121% in AMF inoculated plants (**Figure 3**). In short, significantly greater plant growth performance, as well as MD, generally ranked as *F. mosseae* > *A. scrobiculata* > *R. intraradices* > non-AMF control (**Figure 3**).

### Effects of AMF Colonization on Photosynthetic Variables

**FIGURE 4:**
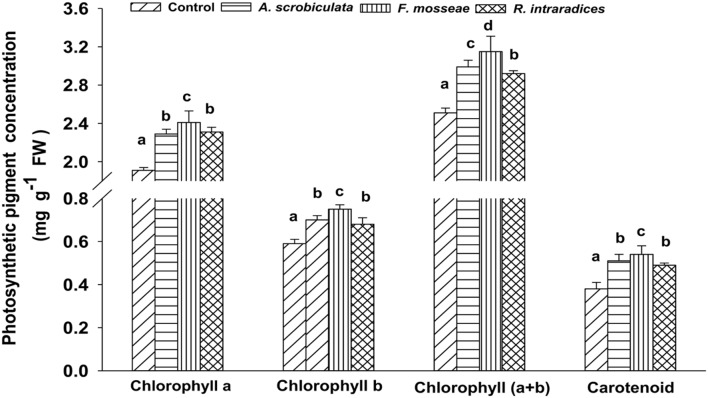
**Effects of mycorrhizal fungal species on the concentrations of leaf chlorophyll a, b, (a+b) and carotenoid of mulberry plants.** Data (means ± SE, *n* = 4) followed by different letters indicate significant differences between AMF treatments at *P* < 0.05. The fresh fifth expanded leaf of each plant was used to determine concentrations of leaf chlorophyll a, b, (a+b) and carotenoid. Forty fresh leaves were used in each treatment. Abbreviations: Control, non-AMF treatment; *A. scrobiculata*, *Acaulospora scrobiculata*; *F. mosseae*, *Funneliformis mosseae*; *R. intraradices*, *Rhizophagus intraradices*.

Concentrations of leaf photosynthetic pigments (chlorophyll a and b, and carotenoid) were significantly higher in all mycorrhizal plants than in non-mycorrhizal plants (**Figure 4**). Among AMF plants, the higher concentration order of tested photosynthetic pigments ranked as *F. mosseae* > *A. scrobiculata* ≈*R. intraradices* for leaf chlorophyll a, leaf chlorophyll b and carotenoid, but under *F. mosseae* > *A. scrobiculata* > *R. intraradices* for leaf chlorophyll (a+b) (**Figure 4**).

**FIGURE 5:**
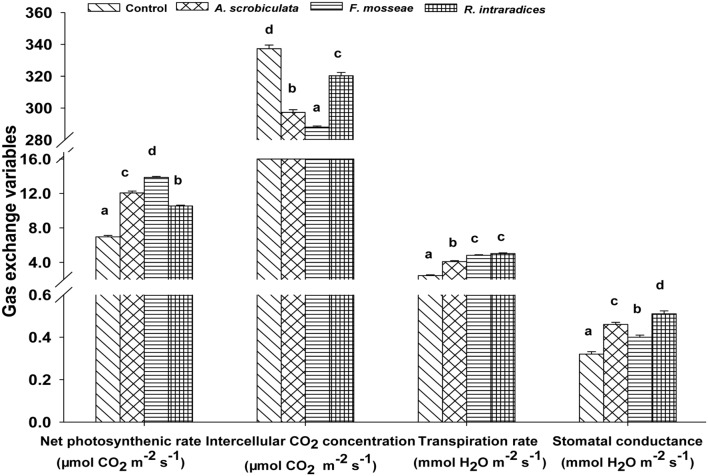
**Effects of mycorrhizal fungal species on net photosynthetic rates, intercellular CO_2_ concentrations, stomatal conductance, and transpiration rates.** Data (means ± SE, *n* = 4) followed by different letters indicate significant differences between AMF treatments at *P* < 0.05. Eighty seedlings from each treatment were randomly selected to detect gas exchange parameters. Abbreviations: Control, non-AMF treatment; *A. scrobiculata*, *Acaulospora scrobiculata*; *F. mosseae*, *Funneliformis mosseae*; *R. intraradices*, *Rhizophagus intraradices*.

Meanwhile, AMF colonization significantly enhanced leaf net photosynthetic rate by 52–99%, stomatal conductance by 24–56%, transpiration rate by 66–104%, while significantly decreased intercellular CO_2_ concentration by 5–15%, compared with non-AMF mulberry plants (**Figure 5**). Among AMF colonized mulberry plants, significantly greater leaf photosynthetic variables, ranked as *F. mosseae* > *A. scrobiculata* > *R. intraradices* for the net photosynthetic rate, *R. intraradices* > *A. scrobiculata* > *F. mosseae* for the stomatal conductance, *F. mosseae* ≈*R. intraradices* > *A. scrobiculata* for the transpiration rate, and *R. intraradices* > *A. scrobiculata* > *F. mosseae* for the intercellular CO_2_ concentrations (**Figure 5**).

### Effects of AMF Colonization on Leaf Quality Variables

**FIGURE 6:**
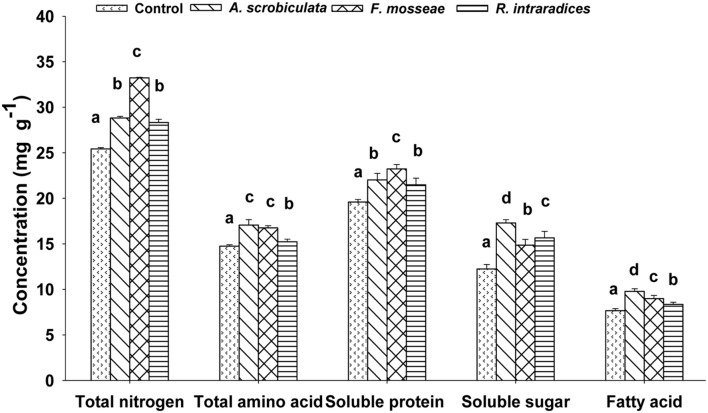
**Effects of mycorrhizal fungal species on leaf total nitrogen (mg g^–1^ DW), total amino acid (mg g^–1^ DW), soluble protein and sugar (mg g^–1^ FW) and fatty acid (mg g^–1^ DW) of mulberry plants.** Data (means ± SE, *n* = 4) followed by different letters indicate significant differences between AMF treatments at *P* < 0.05. The fifth expanded leaf of each plant were used to detect leaf quality. Forty leaves (either fresh or dried) from each treatment were used for the analysis. Abbreviations: Control, non-AMF treatment; *A. scrobiculata*, *Acaulospora scrobiculata*; *F. mosseae*, *Funneliformis mosseae*; *R. intraradices*, *Rhizophagus intraradices*.

Leaf quality including amino acids, total N, soluble sugar and protein, fatty acid of mulberry plants were significantly improved by AMF colonization (**Table 1**; **Figure 6**). Significantly higher concentrations of leaf individual or subtotal essential amino acids, histidine, proline, and total amino acids ranked as *F. mosseae* ≥*A. scrobiculata* > *R. intraradices* (**Table 1**). Significantly higher leaf total N concentrations and soluble protein among AMF colonized mulberry plants ranked as *F. mosseae* > *A. scrobiculata* > *R. intraradices* (**Figure 6**). Significantly higher leaf soluble sugar among AMF colonized mulberry plants ranked as *A. scrobiculata* > *R. intraradices* ≈*F. mosseae*, while significantly higher leaf fatty acid concentrations ranked as *A. scrobiculata > F. mosseae* > *R. intraradices* (**Figure 6**). In addition, significantly higher leaf moisture among AMF colonized mulberry plants patterned as *F. mosseae* (80.56 ± 0.36%) > *A. scrobiculata* (79.11 ± 0.62%) > *R. intraradices* (76.19 ± 0.41%).

**Table 1:** Effects of mycorrhizal fungal species on major amino acid in mulberry leaves.

Treatment	Essential amino acid (mg g^–1^ DW)								His	Pro
	Ile	Leu	Lys	Met	Phe	Thr	Val	Subtotal		
Non-AMF control	0.78 ± 0.01a	1.45 ± 0.02a	0.75 ± 0.01a	0.13 ± 0.01a	0.81 ± 0.01a	0.59 ± 0.01a	0.92 ± 0.01a	5.43 ± 0.07a	0.33 ± 0.02a	1.06 ± 0.02a
*A. scrobiculata*	0.90 ± 0.03b	1.67 ± 0.05b	0.89 ± 0.03b	0.15 ± 0.02b	0.96 ± 0.05b	0.69 ± 0.01b	1.05 ± 0.05b	6.30 ± 0.24b	0.40 ± 0.02b	1.31 ± 0.06c
*F. mosseae*	0.93 ± 0.02b	1.72 ± 0.04b	0.88 ± 0.02b	0.16 ± 0.01b	1.02 ± 0.02b	0.70 ± 0.01b	1.07 ± 0.03b	6.46 ± 0.12b	0.38 ± 0.01b	1.29 ± 0.02c
*R. intraradices*	0.81 ± 0.02a	1.51 ± 0.03a	0.77 ± 0.01a	0.14 ± 0.02a	0.87 ± 0.01a	0.57 ± 0.02a	0.94 ± 0.02a	5.59 ± 0.11a	0.35 ± 0.01a	1.14 ± 0.02b

*Fungal species: A. scrobiculata, Acaulospora scrobiculata; F. mosseae, Funneliformis mosseae; R. intraradices, Rhizophagus intraradices.*

*Data (means ± SE, n = 4) followed by different letters indicate significant differences between AMF treatments at P < 0.05 by the Duncan’s test. Abbreviations; His, Histidine; Ile, Isoleucine; Leu, Leucine; Lys, Lysine; Met, Methionine; Phe, Phenylalanine; Pro, Proline; Thr, Threonine; Subtotal, total essential amino acid; Val, Valine.*

**FIGURE 7:**
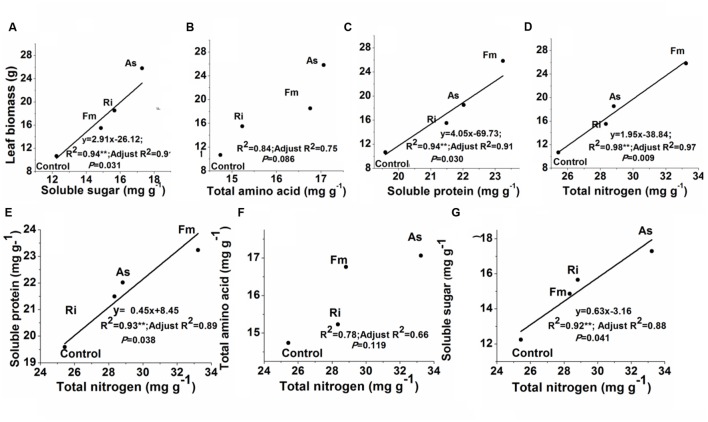
**Relationships between leaf biomass production and soluble sugar (A), total amino acid (B), soluble protein (C) or leaf total nitrogen (D); or between total nitrogen and soluble protein (E), total amino acid (F) or soluble sugar concentration (G) in mulberry plants.** Abbreviations: Control, non-AMF treatment; As, *Acaulospora scrobiculata*; Fm, *Funneliformis mosseae*; Ri, *Rhizophagus intraradices*. **P < 0.05.

Correlations between leaf biomass production and leaf quality were showed in **Figure 7**. Leaf biomass production was significantly related to leaf total nitrogen, soluble protein or soluble sugar (*R*^2^ = 0.94–0.98, *P* < 0.05) (**Figures 7A,C,D**). Meanwhile, leaf total nitrogen concentrations were significantly related to soluble protein or soluble sugar (*R*^2^ = 0.78–0.93, *P* < 0.05) (**Figures 7E,G**). In contrast, either leaf biomass or leaf total nitrogen was not significantly related to total amino acids (*R*^2^ = 0.75–0.78, *P* > 0.05, **Figures 7B,F**).

## Discussion

### AMF Significantly Promotes Growth of Mulberry Plants

In this study, the three AMF species showed different effects on growth of shoot and root in mulberry. Overall *F. mosseae* colonized mulberry seedlings showed the best potential and consistently performed better in respect to plant growth characteristics than other two AMF species of *A. scrobiculata* and *R. intraradices* (**Figures 2** and **3**). However, Lu et al. (2015) found that *R. intraradices* was more efficient than *F. mosseae* in colonizing mulberry roots under greenhouse conditions. Meanwhile, *R. fasciculatus* was more efficient than *Glomus etunicatum* (now *Claroideoglomus etunicatum*) and *F. mosseae* to infect mulberry roots under rainfed lateritic soil conditions (Setua et al., 1999b). As a result, such differences of AMF functioning might be related to AMF species and abiotic factors. In general, the successfully competitive establishment of AMF colonization not only depended on the indigenous mycorrhizal species, but also on the introduced species (Sharma et al., 2005) and its placement in the soil (Hepper et al., 1988). It is well-known that AMF could facilitated mulberry P uptake (Setua et al., 1999a,b) and other macro- and micro-elements uptake (Baqual and Das, 2006; Lu et al., 2015). The uptake function could reduce 75% cost of phosphate fertilization under low soil P levels (Katiyar et al., 1995). Among nine AMF tested (*Acaulospora laevis, G. mosseae, Gigaspora margarita, Glomus caledonicum, G. fasciculatum, G. leptotichum, G. macrocarpum, R. intraradices*, and *Scutellospora calospora*), *G. leptotichum* is the best AM symbiont for 50-day-old nursery teak *(Tectona grandis*) seedlings in terms of P, Zn (zinc), and Cu (copper) nutrition and plant growth (Rajan et al., 2000). The improved nutritional status in mycorrhizal plants contributed to an enhanced plant biomass production (Treseder, 2013; Taylor et al., 2014), resistances of root disease (Al-Askar and Rashad, 2010), salt (Kashyap et al., 2004) and drought (Tang et al., 2013). Moreover, an enhanced root system by mycorrhization could greatly increase the absorption surface and thus nutrient uptake capacity (**Figure 3**). As a result, both AMF species and cultivation management should be taken into account in mulberry plantations.

### AMF Significantly Promotes the Photosynthetic Capacity of Mulberry

#### Leaf Photosynthetic Pigments Are Increased by AMF and Vary with AMF Species

Significantly higher leaf chlorophyll and carotenoid, and photosynthetic rate displayed in the three AMF mulberry plants, and F. mosseae was the best symbiotic associate with the host mulberry (**Figures 1** and **4**). Higher leaf chlorophyll with higher photosynthetic rates under AM associations made it possible to have higher carbon fixation and carbohydrate accumulation (**Figures 4–6**). Concurrently, AMF colonization also enhanced significantly higher carotenoid accumulations, leading to better leaf nutraceutical values of mulberry plants. Leaf carotenoid of mulberry not only acted as a growth-promoting factor for the silkworm larvae (Shimizu et al., 1981), but also played an important role in the larvae’s and adult’s phototactic response and visual sensitivity (Shimizu and Kato, 1978). Meanwhile, the pigmentation development and brilliant color of cocoons were mainly depended on the carotenoid concentration in the mulberry leaves (Tabunoki et al., 2004). Studies also showed that AMF colonization could result in higher concentrations of both leaf chlorophyll and carotenoid in lettuces (Baslam et al., 2013). As a result, the application of AMF species could benefit not only the growth of mulberry, but also the growth and quality of silkworms.

#### Leaf Gas Exchange Capacity Is Enhanced by AMF

The increment of photosynthetic pigments might be due to an effective synergism between transpiration and photosynthesis under mycorrhization (Sheng et al., 2008; Borde et al., 2011). In the present study, photosynthesis, transpiration, and stomatal conductance were significantly greater but intercellular CO_2_ was significantly lower under AMF-inoculated than under non-AMF inoculated mulberry plants. These results from mulberry were consistent with those from maize and alfalfa (*Medicago sativa* L.), regardless of water and salt stress conditions (Zhu et al., 2012; Campanelli et al., 2013), and even under karst rocky desertification area (Chen et al., 2014). The greater stomatal conductance of AM plants implied a lower leaf resistance to moisture diffusion, and ultimately leading to a faster water transportation (Talaat and Shawky, 2014). In addition, an increased leaf area of AMF colonized plants was concomitant with an increase in photosynthetic rate and transpiration rate (**Figure 2B** vs. **Figure 5**). Meanwhile, a potential increase of leaf surfaces with an increase of leaf numbers under mycorrhization could promote plant sunlight capture and thus better photosynthetic production (Adolfsson et al., 2015).

### AMF Significantly Enhances Leaf Quality of Mulberry

#### AMF Inoculation Accelerates Carbohydrate Formation of Mulberry Plants

*Bombyx mori* L. requires essential amino acids, proteins, sugars, and fatty acid from mulberry leaves for its normal growth and silk production (Sengupta et al., 1972). We observed that an increase of leaf soluble sugar in AMF inoculated mulberry plants with higher photosynthetic rates. This observation was consistent with results from other studies, i.e., the inoculation with *F. mosseae* and *R. intraradices* did stimulate soluble sugar production in *Poncirus trifoliate* L. (Wu et al., 2011), *Cyclobalanopsis glauca* (Zhang et al., 2014), and *Jatropha curcas* L. (Kumar et al., 2015). Meanwhile, when leaf moisture was improved and stomas opened wider in mycorrhizal mulberry leaves, greater CO_2_ fixation was then enhanced and carbohydrate accumulation was ultimately increased (Augé et al., 1987). In addition, soluble sugar is a precursor of carotenoids, and an enhanced soluble sugar production could hence increase the carotenoid production (**Figures 4** and **6**; Baslam et al., 2011). Moreover, significantly greater ingestion, digestibility and consumption index were observed in silkworms fed with 80–85% high moisture leaves than with 55–60% low moisture leaves (Rahmathulla et al., 2004). As a result, a higher range of 76–81% leaf moisture under AMF inoculation certified that AMF colonization could improve leaf nutritional quality of mulberry plants. Consequently, the palatability of silkworm to mulberry leaves could be also improved.

#### AMF Inoculation Accelerates Nitrogen Metabolism of Mulberry Plants

The quantity and quality of cocoon shell closely related with concentrations of mulberry leaf N and amino acids, especially methionine, histidine, and threonine. For instance, methionine and histidine are essential for the growth of silkworms, and threonine is crucial for the synthesis of silk protein (Machii and Katagiri, 1991). Our results showed that AMF colonization generally significantly increased total nitrogen, soluble protein, total amino acid, and all seven essential amino acids including methionine, histidine, and threonine, particularly under *F. mosseae* and *A. scrobiculata* (**Figure 6**; **Table 1**). Pentón et al. (2014) reported that inoculation with *Glomus cubense* improved soil N extraction capacity of mulberry and complemented with chemical fertilization. Singh et al. (2010) showed that leaf protein and total amino acid in tea tree were increased by AMF inoculation with an indigenous AMF consortia containing nine AMF species from three genera of *Acaulospora*, *Funneliformis*, and *Glomus*. Govindarajulu et al. (2005) revealed that inorganic N taken up by the AM fungus was incorporated into amino acids and N transportation was from extraradical mycelium to intraradical mycelium and then to plants, which would be a great boost to the nutrition composition and growth of host plants (Azcón et al., 1992). Azcón et al. (1996) also claimed that mycorrhizal colonization increased activity of nitrate reductase and glutamine synthetase involving in N assimilation of the host plant. Therefore, further studies on nitrogen, perhaps also phosphorus nutrition, are guaranteed if mulberry plants could positively response to genetic different AMF species by improving the growth and quality of mulberry hosts.

## Conclusion

Our results showed that inoculation with AM fungi had improved mulberry leaf biomass production and nutritional quality through an enhanced photosynthesis and growth performance. A significantly higher rank of such positive responses of mulberry to AMF species was as follows: *Funneliformis mosseae* > *A. scrobiculata* > *R. intraradices* for plant’s physiological and growth characteristics, and *F. mosseae* ≈ *A. scrobiculata* > *R. intraradices* for leaf quality. Such improvements were relevant to the AMF-induced alterations of leaf carbohydrates and N-containing compounds, essential amino acids and soluble protein in particular. As a result, application of *Funneliformis mosseae* or *A. scrobiculata* in mulberry plantation could be a promising management strategy to promote silkworm cultivation and relevant textile industry in southwest China.

## Author Contributions

X-HY and X-HH conceived and designed the experiments; S-MS, KC, YG, and BL performed the experiments. X-ZH was responsible for the field experiment and provided fund support. G-XL analyzed amino acid data with the help of L-QZ. S-MS and KC wrote the paper. All authors approved the final manuscript.

## Conflict of Interest Statement

The authors declare that the research was conducted in the absence of any commercial or financial relationships that could be construed as a potential conflict of interest.
